# Notum palmitoleoyl-protein carboxylesterase regulates Fas cell surface death receptor-mediated apoptosis via the Wnt signaling pathway in colon adenocarcinoma

**DOI:** 10.1080/21655979.2021.1961657

**Published:** 2021-08-17

**Authors:** Hua Gong, Qiang Niu, Yi Zhou, YUN-Xia Wang, Xuan-Fu Xu, Ke-Zhu Hou

**Affiliations:** a The First Department of General Surgery, Shidong Hospital, Shanghai, P.R. China; b Department of Gastroenterology, Shidong Hospital, Shanghai, P.R. China

**Keywords:** NOTUM, COAD, development, apoptosis, Wnt

## Abstract

Colon adenocarcinoma (COAD) is one of the most common types of malignancy and accounts for >3 million deaths worldwide each year. The present study aimed to evaluate the role of notum palmitoleoyl-protein carboxylesterase (NOTUM) in *in vivo* and *in vitro*, and to identify the relationship between NOTUM and the apoptosis of COAD. Moreover, the present study aimed to investigate whether NOTUM regulated Fas cell surface death receptor (FAS)-mediated apoptosis was affected by the Wnt signaling pathway. Gene expression profiling interactive analysis (GEPIA) was used to predict the potential function of NOTUM. Western blotting and reverse transcription-quantitative PCR were conducted to detect the protein and mRNA expression levels of NOTUM in different tissues or cell lines. The occurrence and development of COAD was detected after NOTUM knockdown lentivirus administration. The apoptosis of COAD was also observed. SKL2001 was applied to examine whether the role of NOTUM was regulated by Wnt. GEPIA analysis demonstrated that NOTUM expression in COAD tumor tissue was higher compared with in normal tissues. Pair-wise gene correlation analysis identified a potential relationship between NOTUM and Wnt. NOTUM protein and mRNA expression levels in colon carcinoma tissues and RKO cells were increased. NOTUM knockdown lentivirus serves a role in inhibiting COAD development by reducing tumor proliferation, reducing tumor size, and increasing the level of apoptosis *in vitro* and *in vivo*. Moreover, NOTUM could increase apoptosis in COAD, which was regulated by FAS, and SKL2001 blocked the progress of apoptosis after NOTUM regulation by NOTUM knockdown lentivirus *in vitro* and *in vivo*. Collectively, the present results suggested that NOTUM may be able to regulate the apoptosis of COAD, and that Wnt may be the down-stream target signaling of NOTUM in apoptosis.

## Introduction

The incidence of colon adenocarcinoma (COAD) demonstrates geographic variation; for instance, the age-adjusted incidence varies by up to 12-fold among different countries [1], which suggests that the development of colonic cancer is difficult to track. COAD develops from specific types of neoplastic polyps in the colonic mucosa via a multistep process, in terms of molecular genetics [2]. COAD has attracted global attention and there are numerous investigations into the development of novel therapies based on an understanding of the mechanisms that induce this disease, as well as to address key obstacles in the available treatments [3].

Apoptosis is a type of programmed cell death that is regulated at the gene level and results in the orderly and efficient removal of damaged cells, including DNA damage or damage that occurs during development [4]. Dysregulation of apoptosis is associated with unregulated cancer cell proliferation, development, progression and resistance to drug therapies [5]. Therefore, therapeutic strategies targeting molecules involved in apoptotic resistance represent a viable approach and may help to overcome the challenges that have resulted in previous ineffective therapeutic methods [6].

Fas cell surface death receptor (FAS) is a member of the tumor necrosis factor receptor superfamily, known as ‘death receptors’, and has been revealed to serve an important role in initiating cell apoptosis [7]. However, the mechanism underlying FAS-mediated apoptosis requires further investigation.

Notum palmitoleoyl-protein carboxylesterase (NOTUM) was first discovered in fruit flies, and it is composed of four exons. When the expression of the NOTUM gene increases, fruit flies are born wingless [8]. The NOTUM gene is involved in the production of vertebrate nerves and heads in the embryo, as well as in the regulation of brain growth in planaria [9]. From fruit flies to humans, NOTUM genes have been identified to function mainly via an association with the Wnt signaling pathway [10]. Recently, NOTUM has been reported to regulate cancer development in numerous tumor types, including invasive urothelial carcinoma of the bladder [11] and colorectal cancer [12]. NOTUM is also involved in abnormal development of the thorax and in apoptosis in *Drosophila melanogaster* [13], and this has prompted researchers to examine whether NOTUM could be employed for the regulation of apoptosis in some diseases. However, the mechanism via which NOTUM regulates apoptosis in COAD is not yet fully understood.

The present study, we aimed to explore the role of NOTUM in COAD and used NOTUM knockdown lentivirus to enhance the apoptotic and development of tumor tissues *in vivo* and *in vitro*. Furthermore, Wnt agonists were used to induce Wnt level and to reverse the apoptosis by regulating NOTUM. The hypothesis of present findings indicated that NOTUM could possibly regulate Fas-mediated apoptosis via the Wnt signaling pathway in COAD. These results may provide novel strategies for COAD treatment by utilizing the mechanism of apoptosis.

## Materials and methods

### Gene expression profiling interactive analysis (GEPIA)

COAD genomics datasets (Ensembl ID: ENSG00000185269.11) were analyzed via the GEPIA website (https://gepia.cancer-pku.cn/), as described in a previous study [1]. The expression profile assay in different digestive system carcinomas, including cholangiocarcinoma, COAD, esophageal carcinoma (ESCA), rectum adenocarcinoma (READ) and stomach adenocarcinoma (STAD), and the isoform analysis in the boxplot for NOTUM expression of COAD were in the ‘profiles and box plot in expression DIY’ module. The pathological stage plot assay was in the ‘stage plot in expression DIY’ module. Survival analysis was in the module of ‘survival analysis’, and pair-wise gene correlation analysis was in ‘correlation analysis’ module.

### Human tumor specimens

For this study, 20 patients (12 male and 8 females; age range, 40–69 years old) who were pathologically diagnosed with COAD were selected at the Digestive Surgery Department in Shidong Hospital (Shanghai, China) between March 2018 and March 2019, and 12 samples (6 male and 6 females; age range, 45–69 years old) of normal tissues collected from around the tumor (para-tumor) were used as the control group. The inclusion criteria were as follows: i) Complete clinical data; ii) diagnosed by the Pathology Department; and iii) medium to low level tubular adenocarcinoma pathologically. The exclusion criteria included: i) Incomplete clinical data; ii) combined with other intestinal tumors; and iii) tumor metastasis. All experimental procedures were approved by the Human Ethics Committee of Shidong Hospital (No.2018041201), and informed written consent was obtained from each subject prior to the study. Table 1 lists the basic information of the patients.

**Table 1.** Clinicopathological features of patients with colon adenocarcinoma

Parameter	Colon adenocarcinoma	Normal tissues
n	20	12
Age, year	56.0 ± 8.0 (40–69)	57.3 ± 6.8 (45–69)
Sex		
Male	12 (60%)	6 (50%)
Female	8 (40%)	6 (50%)
Tumor diameter, cm		
<3	12 (60%)	-
≥3	8 (40%)	-
Tumor side		
Right	2 (10%)	1 (8.33%)
Transverse	4 (20%)	2 (16.67%)
Left	14 (70%)	9 (75%)

### Cell lines and cell cultures

The cell lines used were as follows: NCM460 cells (cat. no. CC-Y1550; EK-Bioscience GmbH; a human colon epithelial cell line), RKO cells (cat. no. CL- 196; Procell Life Science & Technology Co., Ltd.), HCT116 cells (cat. no. CL-0096, Procell Life Science & Technology Co., Ltd.) and SW620 cells (cat. no. CL-0225; Procell Life Science & Technology Co., Ltd.). RKO, HCT116 and SW620 are human COAD cell lines. Cells were cultured at 5% CO_2_ and 37°C with DMEM (Sigma-Aldrich; Merck KGaA), as described in a previous study [14]. Cells were treated with 10 μg SKL2001 (Selleck Chemicals) for 7 days at 37°C for the last part of the experiments *in vitro*.

### Animals

A total of 294 4-week-old healthy male Balb/c mice (weight, 30–40 g), which were supplied by the vivarium of the State Research Center of Virology and Biotechnology, were housed in an appropriate environment under specific pathogen-free (SPF) conditions with a humidity of 60 ± 5%, and were provided unlimited access to food and water. All animals used in this experiment were cared for in strict accordance with the Guide for the Care and Use of Laboratory Animals (NIH Publication No. 85–23, revised 1996) [15]. All experimental procedures were approved by the Animal Ethics Committee of the Shidong Hospital (No.2018041202).

### Reagent and antibodies

The reagent used was as SKL2001 (cat. no. S8320; Selleck Chemicals). The primary antibodies used were as follows: Anti-NOTUM (cat. no. ab106448; Abcam), anti-FAS (cat. no. ab15285; Abcam), anti-BAX (cat. no. ab32503; Abcam), anti-Bcl-2 (cat. no. ab182858; Abcam), anti-cleaved caspase-3 (cat. no. ab49822; Abcam; 1:1,000), anti-cleaved poly (ADP-ribose) polymerase 1 (PARP1; cat. no. ab32064; Abcam; 1:1,000) and anti-β-actin (cat. no. M01263-2; Wuhan Boster Biological Technology, Ltd.; 1:5,000). The secondary antibodies used were conjugated to horseradish peroxidase anti-rabbit IgG (H + L) (cat. no. AS014; ABclonal Biotech Co., Ltd.; 1:5,000) and anti-mouse IgG (H + L) (cat. no. AS003; ABclonal Biotech Co., Ltd.; 1:5,000).

### NOTUM knockdown lentivirus administration

NOTUM knockdown lentivirus (sh-NOTUM), sh-NC lentivirus and control lentivirus were designed and chemically synthesized by Shanghai GenePharma Co., Ltd, along with the transfection reagent. Lentivirus vectors were stored at −80°C and inoculated as described in a previous study [1]. The cell protein or mRNA was extracted, centrifuged and precipitated using Phosphatase inhibitor cocktail B (cat. no. P1086; Beyotime Institute of Biotechnology) and Beyozol (cat. no. R0011; Beyotime Institute of Technology) from 6 well after 72 h. Cells transfected with lentivirus after passage were used to establish the COAD mouse model. The sequences of the lentivirus NOTUM are listed in Table 2.

**Table 2.** Sequence of NOTUM lentivirus

Name	Sequence
sh-NOTUM-361	5ʹ-GCCGGGGTCAGCAGCCGCCT-3’
sh-NOTUM-542	5ʹ-CCTGCGCCTGCACCTCCTAC-3’
sh-NOTUM-964	5ʹ-CCGGGAGCAGCGCGGGGGGC-3’
Negative Control	5ʹ-CGCCTCGACGCCGAGCCGCG-3’
control-shNOTUM	5ʹ-GGGGGAGCAGGAGCGGCCAG-3’

NOTUM, notum palmitoleoyl-protein carboxylesterase; shRNA, short hairpin RNA.

### Subcutaneous xenograft mouse model

Male Balb/c mice (age, 4 weeks) were fed in an SPF environment, and 200 μl 1x10^5^/ml RKO cells with PBS were injected subcutaneously in the back of the hind leg. After 7 days, vital signs and inoculation sites were observed. Mice were treated according to the ‘Laboratory Animal Welfare from the American College of Laboratory Animal Medicine, 2014, Pages 29–38, Chapter 4-Animal Welfare Assessment Considerations’ [16]. The investigators observed the mice daily to ensure animal welfare and to determine the humane endpoints, as described in a previous study [2]. In total, 10 mg/kg SKL2001 was administered to the mice for 7 days at 37°C for the last part of the experiments *in vivo*.

Mice were sacrifice via heart perfusion with 0.9% saline solution. The mice were anesthetized chloral hydrate (400 mg/kg; intraperitoneal), an upper abdominal incision was made, and the heart was exposed. Then, 0.9% saline solution was injected into the apex of heart before cutting open the right auricle and pumped into the systemic circulation.

The experimental groups (n = 6 mice/group) were as follows: Con group (untransfected COAD cells), sh-NOTUM group and control sh-NOTUM group. In total, 96 mice were involved in this experiment. The skin was cut directly, and tumor tissue was collected subcutaneously after modeling for 28 days. Tumor volume was calculated as 0.5 x length x width ^2^ (mm^3^).

### Western blot analysis

Radio-Immunoprecipitation Assay (RIPA) lysis buffer (Beyotime Institute of Biotechnology) containing the protease inhibitor was used to extract total protein from the cells and the tissues, and the supernatant was collected after centrifugation (12,000 x g for 20 min at 4°C). In brief, ~40 μg protein from each tissue sample were subjected to 12% SDS-PAGE and transferred to a PVDF membrane as described in a previous study [3]. All the samples were detected using a ChemiDoc™ Touch Imaging system (Bio-Rad Laboratories, Inc.) and were analyzed using Image Lab 3.0 software (Bio-Rad Laboratories, Inc.).

### Reverse transcription-quantitative (RT-q)PCR

Total RNA was extracted from frozen tissue using TRIzol® (Invitrogen; Thermo Fisher Scientific, Inc.) and reverse transcribed into cDNA using a PrimeScript™ RT Reagent kit with gDNA Eraser (Takara Biotechnology Co., Ltd.) according to the manufacturer’s instructionsas, as described in a previous study [4]. The cDNA was checked using a cocktail of random primers and reverse-transcriptase enzyme. 50 nmol primer sequences for amplification were as follows: NOTUM forward, 5ʹ-CTCCATTTTACAAGCAGCAG-3ʹ and reverse, 5ʹ-GCTCTTTCCTATCCTGTTCA-3ʹ; and β-actin forward, 5ʹ-TGTTTGAGACCTTCAACACC-3ʹand reverse, 5ʹ-CGCTCATTGCCGATAGTGAT-3ʹ (Invitrogen; Thermo Fisher Scientific, Inc.). A total of 40 μl RNA was reverse transcribed into cDNA in 42°C, 55 min and 98°C, 5 min. The PCR reactions were performed with Takara SYBR Premix Ex Taq II (TliRnaseH Plus; Takara Biotechnology Co., Ltd.) on a PCR amplifier in 95°C, 10s, 95°C, 5s, 65°C, 15s and 72°C, 15s in 40 thermocyclings. NOTUM mRNA expression was calculated according to its ratio to β-actin, and the expression levels were calculated using the 2^−∆∆Cq^ method [17].

### Propidium Iodide (PI) staining

Cell viability was assessed using the PI-Hoechst assay (Beijing Solarbio Science & Technology Co., Ltd), according to the manufacturer’s instruction. Cells were observed using fluorescence microscopy at x400 magnification. Images were acquired with a Nikon Eclipse Ni inverted microscope (TE2000; Nikon Corporation).

### Terminal deoxynucleotidyl transferase-mediated dUTP-biotin nick end labeling assay (TUNEL) staining

Apoptotic cells were detected using a TUNEL assay (Beijing Solarbio Science & Technology Co., Ltd.). The TUNEL assay was performed according to the manufacturer’s protocol. Briefly, 5 μl Tdt enzyme (10%) and 45 μl fluorescence-labeled solution (90%) was incubated with the sections for 1 h at 37°C. The section was washed withphosphate belanced solution (PBS) three times and mounted with mounting medium (Sigma-Aldrich; Merck KGaA). The nuclei of the cells were stained with DAPI for 1 min at 37°C. Images were captured using fluorescence microscopy at x400 magnification in 10 randomly selected fields of view [18].

*3-(4,5-Dimethylthiazol-2-yl)-2,5-diphenyltetrazolium bromide (MTT) assay*. After stirring the solution for 30 min, cells in the logarithmic growth stage were inoculated into 96-well plates in groups. Cells with a concentration of 200 ml of 4x10^4^/ml were inoculated into each well as described in a previous study [2]. The absorbance values of the wells were determined using an enzyme-linked immunoassay reader at 490 nm.

### Statistical analysis

Data are presented as the mean ± standard deviation (SD). Data statistical analysis and correlation analysis were performed using GraphPad Prism 6.0 (GraphPad Software, Inc.). Survival analysis was performed using SPSS 19.0 (IBM Corp.). The homogeneity variance and analysis of variance were compared in every group. Differences were analyzed using one-way ANOVA, and multiple comparisons were analyzed using the Sidak test. The survival rates were analyzed using log-rank test and pair-wise gene correlation was analyzed using correlation test. P < 0.05 was considered to indicate a statistically significant difference.

## Results

We aimed to explore the role of NOTUM in COAD and used NOTUM knockdown lentivirus to enhance the apoptotic and development of tumor tissues *in vivo and in vitro*. Furthermore, SKL2001 was used to induce Wnt level and to reverse the apoptosis. The hypothesis was that NOTUM could regulate Fas-mediated apoptosis via the Wnt signaling pathway in COAD.

### Bioinformatic analysis of the role of NOTUM in COAD and other digestive system carcinomas using the GEPIA server

**Figure 1.**
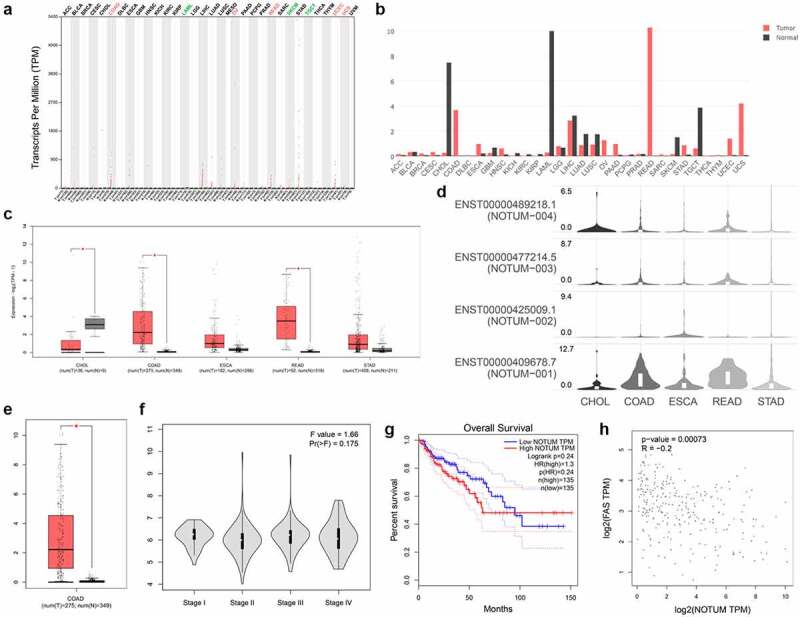
Bioinformatic analysis of the role of NOTUM in COAD and other digestive system carcinomas using the GEPIA serve. (a) Specific quantified value. (b) Expression profile in different cancer types from the GEPIA databases. (c and d) Protein expression profile assay in different digestive system carcinomas. (e) Isoform analysis for NOTUM in COAD, presented as a boxplot. (f) Pathological stage plot assay for NOTUM in COAD. (g) Survival analyses in COAD cancer type. (h) Pair-wise gene correlation analysis of NOTUM and FAS. NOTUM, notum palmitoleoyl-protein carboxylesterase; COAD, colon adenocarcinoma; FAS, Fas cell surface death receptor; T, tumor; N, normal; TPM, transcripts per million

The predicted function of NOTUM in COAD and other digestive system carcinomas from GEPIA and TCGA were presented in Figure 1. As demonstrated in Figure 1a–d, the expression profile and transcripts per million was quantified in different cancer types. NOTUM expression in COAD, ESCA, READ and STAD tumor tissues was significantly higher compared with that in normal tissues, especially in COAD and READ. From the isoform analysis shown in a boxplot (Figure 1e), the NOTUM isoform was over-expressed in COAD, compared with that in normal tissues. The expression of NOTUM in different pathological stages is illustrated in figure 1f, and the difference was not statistically significant among various stages. Patients with COAD with a high expression of NOTUM isoform had a poor prognostic outcome (Figure 1g). Pair-wise gene correlation analysis of NOTUM with FAS demonstrated a potential relationship between NOTUM and FAS (Figure 1h).

### Varying levels of NOTUM expression in different tissues or cell lines

**Figure 2.**
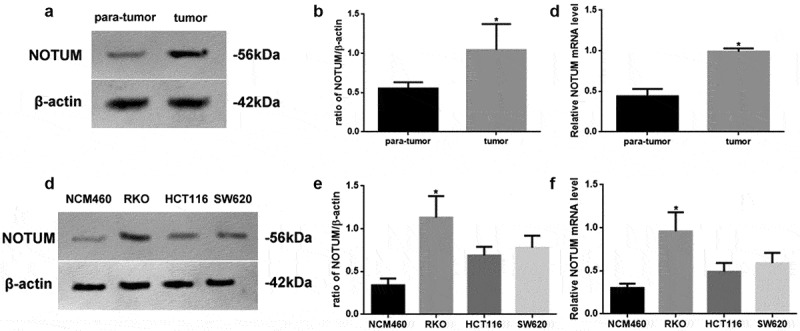
Expression levels of NOTUM in different tissues or cell lines. (a) Western blot analysis of NOTUM expression in tumor and para-tumor tissues, and (b) semi-quantification of results. (c) RT-qPCR assay for mRNA expression of NOTUM in tumor and para-tumor tissues. (d) Western blot analysis of NOTUM expression in NCM460, RKO, HCT116 and SW620 cells, and (e) semi-quantification of results. (f) RT-qPCR assay for mRNA expression of NOTUM in NCM460, RKO, HCT116 and SW620 cells. Protein and mRNA expression levels were normalized to β-actin. n = 6 per group. Data are presented as the mean ± SD. *P < 0.05, vs. para-tumor or NCM460 cells. NOTUM, notum palmitoleoyl-protein carboxylesterase; RT-qPCR, reverse transcription-quantitative PCR

The protein and mRNA levels of NOTUM in different tissues or cell lines were examined using western blot analysis and RT-qPCR (Figure 2). NOTUM protein and mRNA expression levels in colon carcinoma tissues were increased (P < 0.05), compared with those in para-tumor tissue (Figure 2a–c). In addition, NOTUM protein and mRNA expression levels in RKO cells were significantly increased (P < 0.05), compared with other cells (Figure 2d–f). Thus, RKO cells were used as the colon carcinoma model for the follow-up experiments.

### Knockdown of NOTUM could regulate colon carcinoma development and apoptosis in vitro

**Figure 3.**
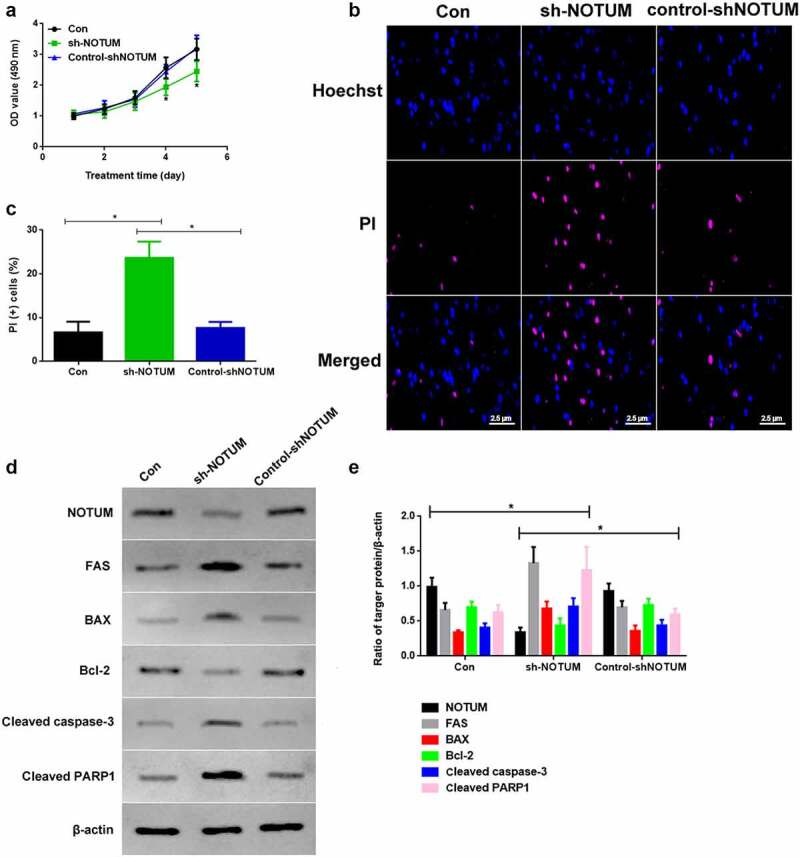
Knockdown of NOTUM regulates colon adenocarcinoma development and apoptosis *in vitro*. (a) Proliferation assay was conducted over 5 days. (b) PI staining assay was performed to examine apoptosis. (c) PI (+) cells assay was performed in Con, sh-NOTUM and control-shNOTUM groups *in vitro*. (d) Western blot analysis of NOTUM, FAS, BAX, Bcl-2, cleaved caspase-3 and cleaved PARP1 expression levels *in vitro*. (e) Semi-quantification of NOTUM, FAS, BAX, Bcl-2, cleaved caspase-3 and cleaved PARP1 expression levels. Protein levels were normalized to β-actin. n = 6 per group. *P < 0.05, Con and control-shNOTUM groups vs. sh-NOTUM group. NOTUM, notum palmitoleoyl-protein carboxylesterase; FAS, Fas cell surface death receptor; PARP1, poly (ADP-ribose) polymerase 1; Con, control; shRNA, short hairpin RNA; OD, optical density

As NOTUM expression is up-regulated in tumors and may be correlated with tumor progression, NOTUM expression was blocked using a NOTUM knockdown lentivirus *in vitro* to examine the effects (Figure 3). The cell proliferation of Con, sh-NOTUM and control-shNOTUM RKO cells was investigated. The absorbance values of the MTT assay on 4th and 5th days in the sh-NOTUM group was significantly lower compared with those in the other two groups (Figure 3a). To investigate the apoptotic function of NOTUM, PI staining was used to observe the changes of the number of the apoptotic cells (Figure 3b). The apoptotic level of cells in the sh-NOTUM group was increased compared with the Con and control-shNOTUM groups (P < 0.05; Figure 3c). In addition, proteins associated with apoptosis were detected via western blotting (Figure 3d). The expression levels of FAS, BAX, cleaved caspase-3 and cleaved PARP1 were increased in the sh-NOTUM group (P < 0.05) compared with the Con and control-shNOTUM groups, while NOTUM and Bcl-2 expression levels demonstrated the opposite trends (Figure 3e).

### Knockdown of NOTUM could regulate colon carcinoma development and apoptosis in vivo

**Figure 4.**
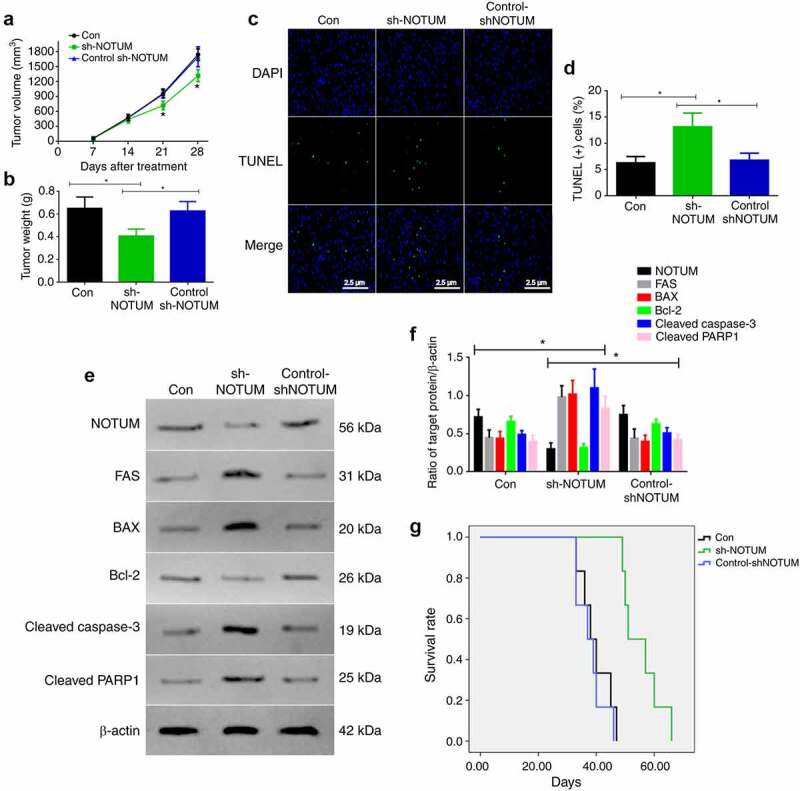
Knockdown of NOTUM regulates colon adenocarcinoma development and apoptosis *in vivo*. (a) Tumor volume over 28 days period and (b) tumor weight at 28 day in the Con, sh-NOTUM and control-shNOTUM mice. (c and d) Immunofluorescence assay of TUNEL staining (magnification, x400) and (d) TUNEL(+) cells. (e) Western blot analysis of NOTUM, FAS, BAX, Bcl-2, cleaved caspase-3 and cleaved PARP1 expression levels *in vivo*. (f) Semi-quantification of NOTUM, FAS, BAX, Bcl-2, cleaved caspase-3 and cleaved PARP1 expression levels. Protein levels were normalized to β-actin. *P < 0.05, Con and control-shNOTUM groups vs. sh-NOTUM group. (g) Survival analysis of Con, sh-NOTUM and control-shNOTUM mice. n = 6 per group. NOTUM, notum palmitoleoyl-protein carboxylesterase; FAS, Fas cell surface death receptor; PARP1, poly(ADP-ribose) polymerase 1; Con, control; shRNA, short hairpin RNA

As the knockdown of NOTUM could regulate colon carcinoma development and apoptosis *in vitro*, this process was also examined *in vivo* (Figure 4). Firstly, tumor volume and tumor weights were measured in the Con, sh-NOTUM and control-sh-NOTUM mice. The tumor volume on 21 and 28 days in sh-NOTUM mice was significantly lower compared with that in the other two groups (P < 0.05; Figure 4a). The tumor weight in sh-NOTUM mice was also decreased (P < 0.05; Figure 4b). Next, in order to investigate the apoptotic function of NOTUM, TUNEL staining was conducted to observe the changes of the apoptotic cells (Figure 4c), and the level of apoptotic cells in sh-NOTUM mice was increased, compared with the Con and control-shNOTUM groups (P < 0.05; Figure 4d). In addition, proteins associated with apoptosis were detected using western blot analysis (Figure 4e). The expression levels of FAS, BAX, cleaved caspase-3 and cleaved PARP1 were increased in the sh-NOTUM group (P < 0.05), compared with their expression in the other groups, and NOTUM and Bcl-2 demonstrated contrary results (figure 4f). Moreover, the survival rate was significantly increased in sh-NOTUM mice compared with the Con and control-shNOTUM groups (P < 0.05; Figure 4g).

### NOTUM regulates FAS-mediated apoptosis via the Wnt signaling pathway

**Figure 5.**
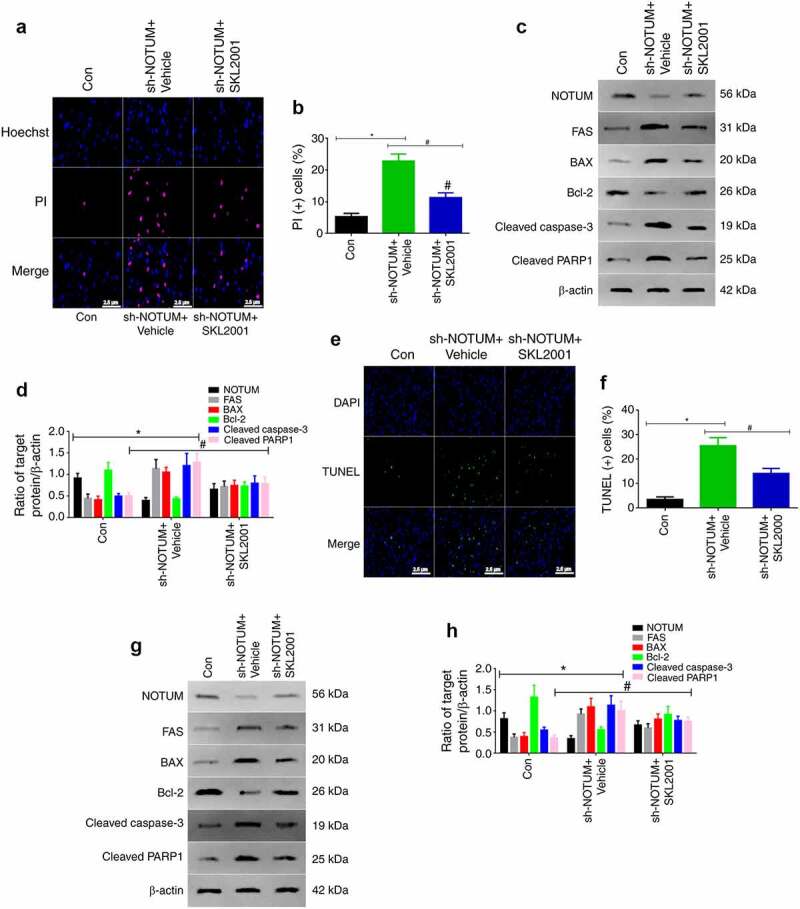
NOTUM regulates FAS-mediated apoptosis via the Wnt signaling pathway (a) PI staining assay of apoptosis and (b) quantification of PI (+) cells in Con, sh-NOTUM + Vehicle and sh-NOTUM + SKL2001 mice. (c) Western blot analysis of NOTUM, FAS, BAX, Bcl-2, cleaved caspase-3 and cleaved PARP1 expression levels *in vitro*. (d) Semi-quantification of NOTUM, FAS, BAX, Bcl-2, cleaved caspase-3 and cleaved PARP1 expression levels. (e) Immunofluorescence assay of TUNEL staining (magnification, x400) and (f) number of TUNEL(+) cells in Con, sh-NOTUM + Vehicle and sh-NOTUM + SKL2001 mice. (g) Western blot analysis of NOTUM, FAS, BAX, Bcl-2, cleaved caspase-3 and cleaved PARP1 expression levels *in vivo*, and (h) semi-quantification of results. Protein levels were normalized to β-actin. n = 6 per group. *P < 0.05, sh-NOTUM + vehicle group vs. Con group; ^#^P < 0.05, sh-NOTUM + SKL2001 group vs. sh-NOTUM + vehicle group. NOTUM, notum palmitoleoyl-protein carboxylesterase; FAS, Fas cell surface death receptor; PARP1, poly(ADP-ribose) polymerase 1; Con, control; shRNA, short hairpin RNA

To investigate whether NOTUM is able to regulate Fas-mediated apoptosis via the Wnt signaling, Wnt expression was increased by using SKL2001, which is a Wnt agonist (18). In the experiment, SKL2001 was used to enhance Wnt expression, and apoptosis in cells was examined via western blot analysis and immunofluorescence *in vitro* and *in vivo* (Figure 5). As presented in Figure 5a and b, compared with the sh-NOTUM + Vehicle group, the level of apoptotic cells was significantly decreased in the sh-NOTUM + SKL2001 group *in vitro* (P < 0.05). The expression levels of FAS, BAX, cleaved caspase-3 and cleaved PARP1 in the sh-NOTUM + SKL2001 group were also decreased (P < 0.05), compared with the sh-NOTUM + Vehicle group. Moreover, the opposite results were obtained for NOTUM and Bcl-2 expression levels (Figure 5c and d). *In vivo*, as presented in Figure 5e and f, compared with the sh-NOTUM + Vehicle group, the level of apoptotic cells was significantly decreased in the sh-NOTUM + SKL2001 group (P < 0.05). The expression levels of FAS, Bax, cleaved caspase-3 and cleaved PARP1 in the sh-NOTUM + SKL2001 group were also decreased (P < 0.05), compared with the sh-NOTUM + Vehicle group. Moreover, the opposite results were obtained for NOTUM and Bcl-2 expression levels (Figure 5g and h).

## Discussion

Colorectal cancer is the third most common cancer type and the fourth most common cause of mortality globally, accounting for ~12 million new cases and 600,000 mortalities per year [19]. Previous studies have provided evidence for the ongoing discussion of the requirement to performed molecular tumor analysis in all patients with COAD who received adjuvant chemotherapy [20,21].

Although blood-based protein biomarkers are already utilized in clinical settings, including P53, isocitrate dehydrogenase 1 and Bcl-2, they cannot be used alone to screen or diagnose cancer as the levels of these markers can be abnormal for reasons other than cancer [22]. Thus, there is an urgent requirement for novel biomarkers and molecular targets with relatively high sensitivity and specificity.

NOTUM is a 56-kDa evolutionarily conserved secreted deacylase that is associated the Wnt signaling pathway [23]. NOTUM is overexpressed in hepatocellular carcinoma [24], gastric cancer [25], invasive urothelial carcinoma of the bladder [11] and colorectal cancer [12]. These findings suggest that NOTUM serves a role in multiple cancer types. In order to investigate the potential role of NOTUM in COAD, GEPIA databases were used to predict the potential function of NOTUM in the present study. The current results suggested that NOTUM expression in COAD, ESCA, READ and STAD tumor tissues was higher compared with that in normal tissues, especially in COAD and READ. Moreover, patients with COAD with high expression of the NOTUM isoform had a poor prognostic outcome. Pair-wise gene correlation analysis identified a potential relationship between NOTUM and FAS. Thus, these results indicated that NOTUM may serve a role in apoptosis in COAD.

In the present study, western blot analysis and RT-qPCR were used to analyze the protein and mRNA expression levels of NOTUM in different tissue or cell lines, and it was found that NOTUM expression was increased. This suggests that NOTUM may be involved in the development and progression of colon cancer.

FAS stimulates the initiation of apoptosis, which leads to the recruitment of FAS-associated death domain proteins and self-activates caspase-8, ultimately leading to apoptosis [26]. To investigate the relationship between NOTUM and FAS, PI and TUNEL staining were conducted, and apoptotic-related protein expression levels were detected via western blotting after NOTUM knockdown lentivirus administration *in vitro* and *in vivo*. The apoptotic cell levels were increased in the sh-NOTUM group. In addition, NOTUM knockdown lentivirus administration improved proliferation, tumor volume, tumor weight and survival rate. These results demonstrated that low levels of NOTUM can significantly inhibit the proliferation and metastasis of colon cancer cells by regulating FAS-mediated apoptosis, as well as reduce the tumor volume and ultimately improve the prognosis.

The Wnt signaling pathway is associated with cell differentiation, polarization and migration during development [27]. Previous studies have reported that the Wnt signaling pathway is a key pathway involved in various processes of colon cancer [28]. To date, studies have examined the roles and mechanisms of Wnt signaling in regulating cell apoptosis, stimulating angiogenesis and maintaining highly resistant CSCs [29,30]. The Wnt signaling pathway has recently been revealed to be associated with NOTUM [31]. However, the mechanism via which NOTUM regulates apoptosis via the Wnt signaling pathway in COAD is not fully understood. To address this issue, the present study used Wnt agonists to increase Wnt signaling activity. The present results demonstrated that the Wnt agonists reversed the progress of apoptosis by NOTUM regulation. Thus, it was suggested that NOTUM regulated Fas-mediated apoptosis via Wnt signaling.

However, the present study also has several limitations; firstly, the direct relationship between NOTUM and Fas was not analyzed using co-immunoprecipitation, thus it was not fully proved that NOTUM and Fas bind. Secondly, for technical reasons, flow cytometric analysis of apoptosis was not performed. Lastly, the mechanism of the signaling pathway should be further investigated.

## Conclusion

The present findings indicated that NOTUM knockdown significantly activated apoptotic function and inhibited the development of COAD by modulating FAS and prolonging the survival time of mice. Collectively, these results may provide novel strategies for COAD treatment by utilizing the mechanism of apoptosis.

## Data Availability

The datasets used and/or analyzed during the current study are available from the corresponding author on reasonable request.

## Highlights

1. NOTUM expression is up-regulated in COAD and may be correlated with tumor progression.
2. NOTUM regulates FAS-mediated apoptosis in COAD.
3. NOTUM regulates apoptosis via the Wnt signaling pathway in COAD.
